# A 3D Analysis of Cleared Human Melanoma

**DOI:** 10.3390/biomedicines10071580

**Published:** 2022-07-02

**Authors:** Vicente Llorente, Daniel Sanderson, Alejandro Martín-Gorgojo, Rafael Samaniego, Manuel Desco, María Victoria Gómez-Gaviro

**Affiliations:** 1 Instituto de Investigación Sanitaria Gregorio Marañón (IiSGM), Doctor Esquerdo 46, 28007 Madrid, Spain; vllorente@hggm.es (V.L.); dsanderson@hggm.es (D.S.); alejandromartingorgojo@gmail.com (A.M.-G.); confocal@hggm.es (R.S.); 2 Servicio de ITS/Dermatología, Sección de Especialidades Médicas, Ayuntamiento de Madrid, 28006 Madrid, Spain; 3 Departamento de Bioingeniería e Ingeniería Aeroespacial, Universidad Carlos III de Madrid, 28911 Madrid, Spain; 4 Centro de Investigación Biomédica en Red de Salud Mental (CIBERSAM), 28029 Madrid, Spain; 5 Centro de Investigaciones Cardiovasculares (CNIC), Melchor Fernández Almagro, 28029 Madrid, Spain

**Keywords:** melanoma, vasculature, 3D imaging, tissue clearing, SPIM, light-sheet microscopy

## Abstract

Cutaneous melanoma is one of the most aggressive and deadliest cancers in human beings due to its invasiveness and other factors. Histopathological analysis is crucial for a proper diagnosis. Optical tissue clearing is a novel field that allows 3D image acquisition of large-scale biological tissues. Optical clearing and immunolabeling for 3D fluorescence imaging has yet to be extensively applied to melanoma. In the present manuscript, we establish, for the first time, an optical clearing and immunostaining procedure for human melanoma and human cell line-derived melanoma xenograft models using the CUBIC (clear, unobstructed brain imaging cocktails) technique. We have successfully cleared the samples and achieved 3D volumetric visualization of the tumor microenvironment, vasculature, and cell populations.

## 1. Introduction

Melanoma is a high-risk skin cancer with the potential to metastasize; cutaneous malignant melanomas are highly invasive and are frequently resistant to therapy. While only accounting for 1% of all skin cancer cases, it makes up the majority of skin cancer-induced deaths [1]. The probability of developing melanoma is 3.7% for men and 2.5% for women in the United States. More than 150,000 new cases of melanoma were reported throughout 2020 [2], and it is predicted to cause 7650 deaths during 2022 [1]. Histopathological diagnosis is crucial for tumor detection in the first stages of the neoplasm, but its sensitivity is sometimes limited, and alternatives are required.

Analysis of the tumor microenvironment is essential for understanding cancer processes. Specifically, the vasculature is a crucial dynamic marker in tumors and metastases, and its visualization in deeper regions could help improve diagnosis. The analysis of vasculature density, vessel morphology, and distribution concerning target cell populations and similar parameters could advance the study of cancer staging and tumoral cell marker distribution, providing access to novel targets of therapeutic assessment, such as immune infiltrates [3]. In this regard, recent studies have shown that 3D optical imaging and rendering techniques are valuable tools to explore anatomical and functional features of the vasculature [4].

Optical tissue clearing (OTC) techniques are an emerging technology used for 3D imaging of large-scale biological tissues [5,6]. These techniques increase the transparency of the sample by reducing internal light scattering and removing absorptive chromophores, enabling deeper image acquisition. While the basic concept was described over a century ago [7], there has been renewed interest in the technique as a result of recent developments in imaging and microscopy techniques, including light-sheet fluorescence microscopy (LSFM) [8].

Several tissue clearing methods have been developed based on different physicochemical properties and are usually classified into three main “families”: hydrophobic, hydrophilic, and hydrogel-based [9].

Hydrophobic methods were the first to be developed and act by replacing water-based fluids with high Refractive Index (RI) organic solvents. A popular hydrophobic reagent is benzyl alcohol/benzyl benzoate (BABB) [10,11].

Hydrophilic, or aqueous, solutions were developed to solve the organic solvent’s shortcomings and achieve biocompatibility, biosafety, and epitope preservation for immunohistochemistry (IHC) assays. Simple immersion methods place the sample in an aqueous solution of a high-RI solvent (RI > 1.45) to gradually replace the existing fluids and clear the sample. As a low-viscosity liquid, 2,3-thiodiethanol (TDE) can be dissolved at varying concentrations to achieve different RI values. More advanced hydrophilic methods remove lipids and reduce the RI of the sample. Some induce a partial denaturation of hydrophobic proteins to homogenize RIs. The CUBIC (clear, unobstructed brain imaging cocktails) protocol [12] uses detergents to achieve lipid removal and urea/glycerol to hydrate and preserve fluorescence. CUBIC is widely applied as the gold standard for many biological tissues [13,14,15].

The skin has been optically cleared with different reagents, such as disaccharides and hyaluronic acid [16,17,18,19], in combination with various optical techniques [18,20,21,22,23]. In dermatology, optical tissue clearing protocols have been used, in combination with optical coherence tomography (OCT), for cross-sectional imaging to visualize cellular patterns [24]. The diagnosis of cutaneous melanoma and non-melanoma skin cancers using OCT has been widely discussed [25,26,27]. OCT and white-light diffuse reflectance spectroscopy have been used to visualize cleared murine melanoma in a preclinical model using optical clearing agents [28]. In vivo cutaneous microvascular imaging, carried out with dynamic-OCT, may help diagnose different skin cancers [29]. In vivo visual monitoring of skin microvasculature has been proposed for the early diagnosis of diabetes [30,31]. Thus, even though the skin has been a target for optical clearing and imaging, human melanoma has not been cleared before. The main difficulty when dealing this kind of sample is the strong optical absorption caused by melanin pigmentation, limiting most studies to the surface of the tumor.

Histological analysis of skin biopsies is the primary method to establish a melanoma diagnosis [32]. However, it involves physical slicing of the sample, which implies the consequent loss of 3D structural information. Most samples provided by pathological anatomy services are provided sliced, and therefore access to complete human melanoma samples is not simple. As an alternative, xenograft models may be used, as they are suitable and representative models of different tumors dynamics [33]. Human cell line-derived xenograft mouse models include relevant physiological components absent from in vitro models and establish interactions with the murine tissues that allow the study of their dynamics [34]. They can be considered as the next step in the evaluation of therapeutic strategies with respect to in vitro models [34], and they have been used to study different melanoma dynamics [35,36]. Additionally, they are easier to acquire, develop, and analyze in the absence of actual human samples, with intact 3D structure.

In this paper, we explore, for the first time, the applicability of different clearing methods on human melanoma and xenograft melanoma model samples, and we employ a variant of CUBIC Reagent 1, named Reagent 1-A, to better preserve the fragile structure of human melanoma. We use these methods, in combination with 3D fluorescence microscopy, as a potential new technique for the improvement of melanoma study.

## 2. Materials and Methods

### 2.1. Melanoma Xenograft Model

BALB/cOlaHsd-Foxn1nu mice (Harlan-Laboratories, Santiga, Spain), maintained under specific pathogen-free conditions, were subcutaneously inoculated with 10^6^ A375 melanoma cells (left flank), grown in RPMI-1640 medium (Gibco, Alcobendas, Spain), supplemented with 10% fetal calf serum (FCS, Sigma-Aldrich, Madrid, Spain). After 13 days, mice were sacrificed, and tumors were extracted. The procedures were approved by the hospital’s Ethics Committee for Drug Research (Comité Ético de Investigación con Medicamentos, CEIm) (Acta 04/2022, 4 April 2022).

### 2.2. Human Melanoma

Human primary desmoplastic melanoma was biopsied from a stage IV patient undergoing surgical treatment. The sample was embedded in paraformaldehyde (PFA) 4% in Phosphate-Buffered Saline 1× (PBS), overnight (o.n.), at 4 °C and then washed with PBS 3 × 15 min at room temperature (RT).

### 2.3. Sample Clearing and Immunostaining

Samples were cleared with one of the following methods: CUBIC, BABB, or TDE. Each method is briefly explained below. A more detailed review on their properties, advantages, and limitations can be found in the work of authors of [19].

#### 2.3.1. CUBIC

The CUBIC tissue clearing protocol consists of two steps: an initial removal of lipids (as the main contributors to opacity and scattering), followed by the homogenization of RI values by immersion in Reagent 1 (R1) and Reagent 2 (R2), respectively. As the sample only remains transparent while immersed in Reagent 2, IHC assays are performed between both steps. We introduced Reagent 1-A as an alternative to Reagent 1 [37], as it showcases better preservation of fluorescent proteins and an increased clearing power; however, it requires potentially longer incubation times due to a slower action. The duration of the different steps was based on our own modifications of the original CUBIC and CUBIC 1-A protocols, as no melanoma-specific variant of CUBIC has been described before.

Reagent 1 was prepared by mixing 3.5 mL of distilled water (40% by wt), 2.5 g of urea (25% by wt), 2.5 g of N,N,N′,N′-tetrakis (2-hydroxy-propyl) ethylenediamine (20% by wt), and 1.5 g of Triton X-100 (15% by wt). Reagent 1-A was prepared by mixing 3.5 mL of distilled water (75% by wt), 2.5 g of urea (10% by wt), 2.5 g of N,N,N′,N′-tetrakis (2-hydroxy-propyl) ethylenediamine (5% by wt), 1.5 g of Triton X-100 (10% by wt), and NaCl (25 mM). Reagent 2 was prepared by mixing 1.5 mL of distilled water (15% by wt), 1 g of triethanolamine (10% by wt), 5 g of sucrose (50% by wt), and 2.5 g of urea (25% by wt). (Table 1).

Human melanoma samples were cleared by immersion in R1-A containing 4′,6-diamidino-2-phenylindole (DAPI) (1:2000) (Merck, Darmstadt, Germany) for 3 days in a shaker at 80 rpm at 37 °C for thin (1–2 mm) sections and 6 days for larger pieces (>~1 cm). These time windows were adjusted based on the sample clearing progress and our prior experience using the CUBIC protocol.

For the IHC assay, the samples were washed 3 times for 20 min with PBS at room temperature (RT). They were subsequently incubated with 0.02 mg/mL of anti-CD31 (ab28364, Abcam, Cambridge, UK) or Factor VIII (Agilent, Santa Clara, CA, USA), labelling the vasculature with 0.25 μg/mL of DAPI in a shaker at 80 rpm at 37 °C for 24 h for thin sections and for 4 days for larger pieces. The tissue was washed 3 times for 20 min each with PBS-T (Triton 0.1% in PBS) at RT and incubated with 3.3 μg/mL of the secondary antibody and 0.25 μg/mL of DAPI at 37 °C for 24 h for thin sections and for 4 days for larger pieces.

**Figure 1:**
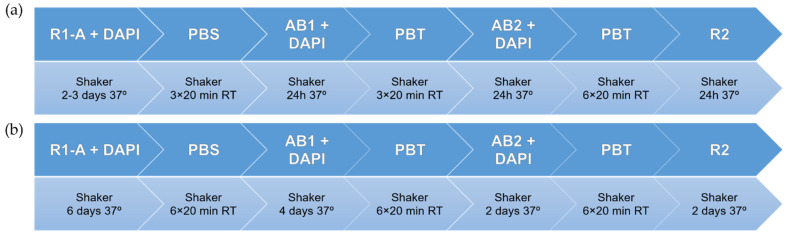
CUBIC 1-A clearing and immunohistochemistry (IHC) staining protocols for (**a**) human melanoma slices and (**b**) larger human melanoma pieces.

The samples were then washed with PBS-T 6 times, for 20 min each, at RT and immersed in CUBIC-clearing reagent-2 (R2) on a shaker at 80 rpm and 37 °C. The protocols are sketched in Figure 1.

**Figure 2:**
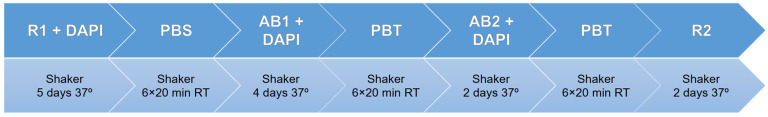
CUBIC clearing and IHC staining protocol for melanoma xenograft model samples.

We used a similar protocol for the xenograft samples, but taking the original version of Reagent 1 instead of the 1-A variant, to assess its effectiveness and shorten the protocol by roughly 1 day. The different steps are sketched in Figure 2.

#### 2.3.2. Benzyl Alcohol/Benzyl Benzoate (BABB)

The BABB protocol requires an initial de-hydration step to remove water from the samples, followed by immersion in the main reagent. Protocol duration depends on the sample size and tissue type, and while it is simple and effective, it quenches fluorescence, thus rendering it incompatible with IHC assays [38]. Therefore, we only used BABB as a reference of clearing power.

**Figure 3:**
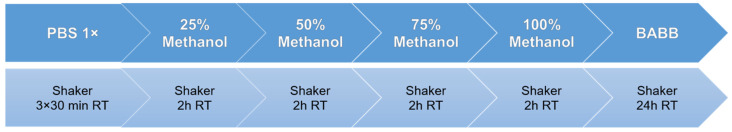
Benzyl Alcohol/Benzyl Benzoate (BABB) clearing protocol.

The protocol is sketched in Figure 3. The samples were first washed with PBS 3 × 30 min, then dehydrated with 2 h incubations in increasing concentrations of methanol in deionized water (25% -> 50% -> 75%), up to 100% pure methanol, o.n. Two parts of benzyl alcohol were mixed with one part of benzyl benzoate to obtain the clearing solution, and incubation was performed o.n. at RT in darkness. The whole process was performed with glass recipients, as BABB can dissolve polystyrene.

#### 2.3.3. 2,3-Thiodiethanol (TDE)

Due to the limited availability of human melanoma samples, we only applied the TDE clearing protocol to melanoma xenograft model samples.

TDE solutions were prepared by mixing 97% volume of TDE with 3% PBS. We used PBS rather than water to better preserve the tissue during clearing and to maintain a neutral pH. We chose this specific concentration of TDE to obtain a high RI value (~1.52), while maintaining a stable pH.

**Figure 4:**
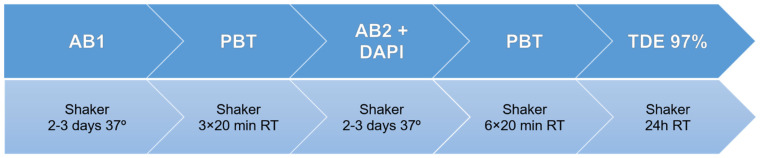
2,3-thiodiethanol (TDE) clearing and IHC staining protocol.

Samples only remain transparent while they are immersed in TDE; this requires the performance of the IHC assays prior to TDE clearing. The samples were incubated with 0.02 mg/mL of anti-Factor VIII in a shaker at 80 rpm at 37 °C for 2–3 days. The tissue was washed 3 times for 20 min with PBT at RT and incubated with 3.3 μg/mL of the secondary antibody and 0.25 μg/mL of DAPI at 37 °C for 2–3 days. The samples were washed with PBS for 10 min and immersed in the TDE solution o.n. The protocol is sketched in Figure 4.

### 2.4. Sample Transparency Assessment

To measure the transparency of the cleared human melanoma and xenograft model, we used a Nikon Eclipse E800 (Nikon, Minato, Tokyo, Japan) bright-field microscope. The sample was illuminated from below, and the transmitted light was captured with a Nikon Plan UW 2× objective (NA: 0.06, WD: 7.5 mm) (Nikon, Minato, Tokyo, Japan). A Nikon DXM1200F digital camera (Nikon, Minato, Tokyo, Japan) mounted above the sample was used to acquire the image. We obtained two types of images: without the sample (representing the base measurement, ***I*_0_**) and with the sample (representing the measurement, ***I***). We applied the Beer–Lambert law to calculate the transparency in terms of the attenuation coefficient ***µ***:

$$I=I_{0}e^{-\mu \;L}$$

***I*** being the intensity of the transmitted light through the sample, ***I*_0_** the incident light intensity, ***µ*** the absorption coefficient, and ***L*** the sample thickness traversed by the light in its path to the camera. The absorption coefficient is calculated as:

μ=lnII0L

A higher absorption coefficient implies a lower amount of light transmitted and thus, a higher opacity.

We implemented the algorithm in a MATLAB^®^ script that randomly selects three small regions of the image (15% of the full image size for each region), computes its attenuation coefficients, and averages them. We acquired three images for each sample and executed the script 15 times on each image, thus obtaining 3 samples/run × 15 runs/image × 3 images/sample = 135 individual measurements for each sample, averaged into a single value.

### 2.5. Hematoxylin and Eosin Staining

For hematoxylin and eosin (H&E) staining, the tissue was immersed in 96% alcohol for 60 min for a total of 3 times. The tissue was then immersed in 99% alcohol for 60 min for a total of 3 times, then twice in isoparaffin for 60 min and twice in paraffin for 60 min, then allowed to cool down. A microtome was used to cut the block into 4 µm sections, and standard H&E staining was then carried out.

H&E conventional microscopy images were acquired with a Nikon Eclipse E800 (Nikon Instruments Inc., Tokyo, Japan) bright field microscope with a Nikon Plan UW 2×/0.06 WD 7.5 objective (Nikon Instruments Inc., Tokyo, Japan) and connected to a Nikon DXM1200F digital microscopy camera (Nikon Instruments Inc., Tokyo, Japan).

### 2.6. Transmission Electron Microscopy

Transmission electron microscopy (TEM) images were acquired using a JEOL JEM—100SX Transmission Electron Microscope (JEOL, Ltd., Tokyo, Japan) from the pathology department of Hospital General Universitario Gregorio Marañón.

### 2.7. Confocal Microscopy

High-resolution images of the samples were acquired with a Leica TCS SPE inverted Confocal Microscope (Leica Microsystems, Germany) using ASC APO 10×/0.30 DRY and ACS APO 20×/0.60/IMM objectives. The 20× images required glycerol immersion.

### 2.8. Selective Plane Illumination Microscopy

3D stacks were acquired with a custom-built selective plane illumination microscope (SPIM). The illumination objective used to focus the laser light sheet onto the focal plane was an infinity-corrected 5× long working distance objective (NA: 0.14, WD: 34 mm, depth of focus (DF): 14 μm) (Mitutoyo Corporation, Japan). Two other infinity-corrected long-working-distance magnification objectives were used for detection: a 2× lens (NA: 0.055, WD: 34 mm and DF: 91 μm) (Mitutoyo Corporation, Japan) and a 5× objective (NA: 0.055, WD: 34 mm and DF: 91 μm) (Mitutoyo Corporation, Japan).

The microscope uses a system of mirrors to direct the laser beam to a cylindrical lens that converts the beam into a light sheet and an illumination objective to focus the sheet on the sample, which is suspended in a glass cuvette. The sample is moved on the z-axis with the help of a high-precision step motor (Zaber, Canada) to acquire images plane-by-plane. The emitted fluorescence is filtered and captured by a Neo 5.5 sCMOS camera (Andor, UK) with 2560 × 2160 active pixels and a physical pixel detector size of 6.5 μm × 6.5 μm. Additional details on the system can be found in the research presented by the authors of [13].

Image acquisition with SPIM is controlled by custom-made software. After placing the sample in the field of view (FOV), the software allows the user to program a sequence of excitation lasers, their respective emission filters, and stage movements. Furthermore, the user can set parameters, such as excitation laser attenuation (power), camera exposure time, initial and final z-positions, and spacing between consecutive acquisitions.

### 2.9. Image Visualization and Processing

Images were processed using the ImageJ Open-Source software and its extension Fiji [39]. Image stacks were filtered with a 3 × 3 median filter to remove salt and pepper noise, when needed. Brightness and contrast were linearly adjusted. In-house developed software was used to remove stripe artefacts and align the lasers. The 3DSlicer software was used for 3D volume rendering [40].

## 3. Results and Discussion

### 3.1. CUBIC and BABB Clearing of Human Melanoma

**Figure 5:**
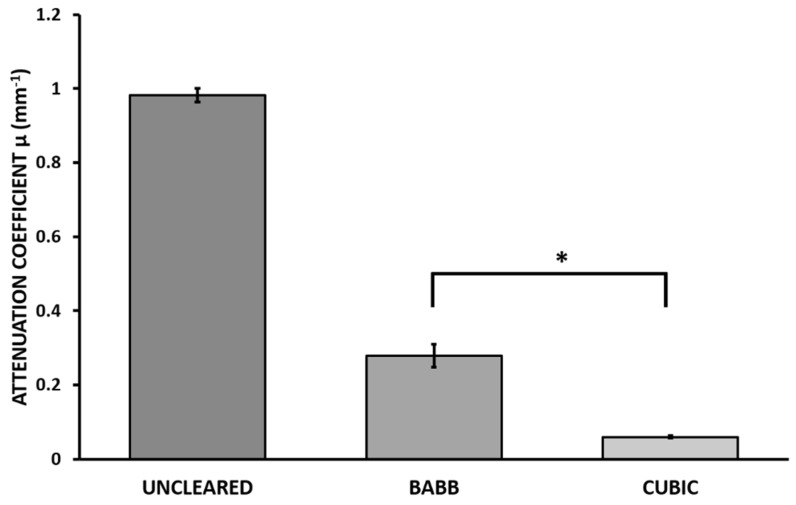
Attenuation coefficients µ (mm^−1^) for each of the images obtained from uncleared, post-BABB incubation, and post-CUBIC 1-A incubation human melanoma samples (data represent mean ± SEM, * *p* < 0.001, one-way ANOVA plus Bonferroni post-test).

In this project, we evaluated whether CUBIC-1A could be an effective method to clear human melanoma. BABB was used as a gold standard to compare the clearing efficiency of CUBIC and TDE. Transparency was measured and calculated using the Beer–Lambert law (Figure 5).

In terms of transparency, both methods successfully cleared the samples, with CUBIC 1-A showing a significant reduction in the attenuation coefficient compared to BABB. However, it required longer times to achieve maximum transparency. Therefore, the appropriate method will depend on the experimental requirements, needing to balance clearing efficiency and protocol duration. The incubation periods for CUBIC 1-A were adjusted to provide the optimal transparency by qualitative observation of the transparency of the samples and the turbidity of R1 caused by floating (extracted) lipids.

Other tissue clearing methods are incompatible with IHC assays, require longer protocols, or include difficult to handle (and expensive) reagents. CUBIC 1-A is an inexpensive and simple clearing method that presents IHC compatibility and preserves endogenous fluorescence and tissue structure.

The main limitation of this study was the low number of human melanoma samples available, which reduced the statistical power of the experiments and impeded clearing with TDE.

### 3.2. CUBIC, TDE, and BABB Clearing of Melanoma Xenograft Model

**Figure 6:**
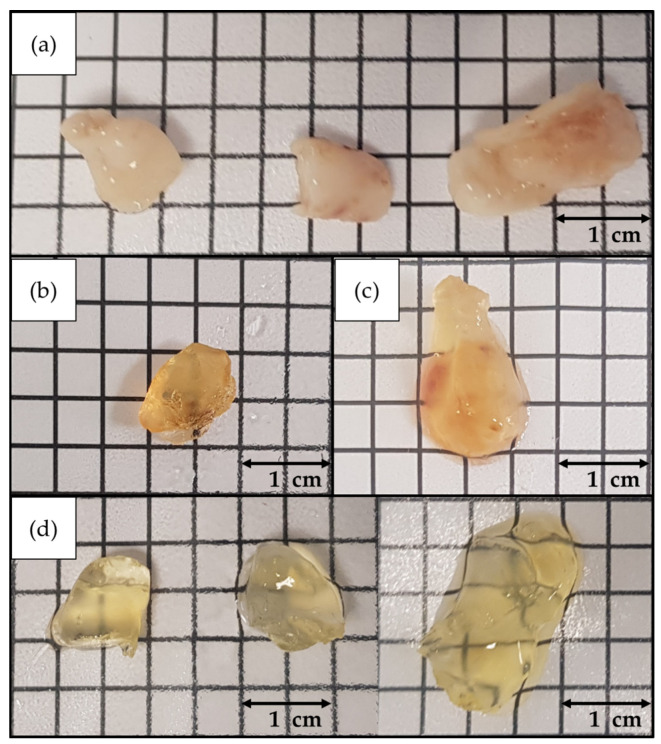
Cell line-derived melanoma xenograft model samples (**a**) before starting the tissue clearing protocols, (**b**) after completing the BABB protocol, (**c**) after completing the TDE protocol, (**d**) after completing the CUBIC protocol.

**Figure 7:**
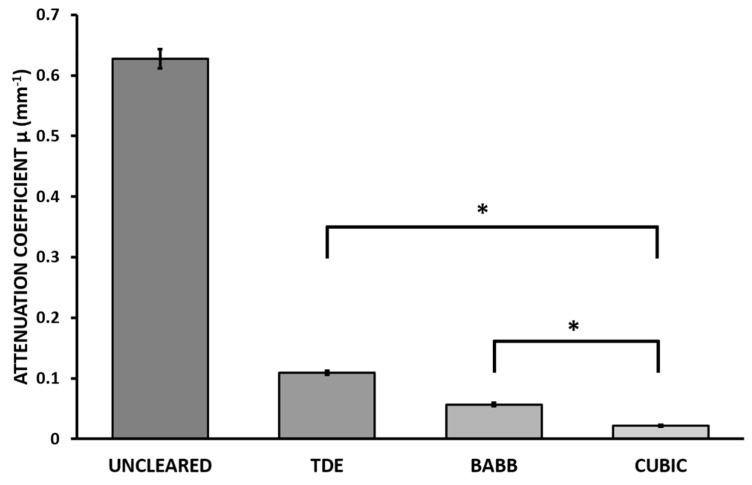
Final average attenuation coefficient values µ for each of the images obtained from uncleared, post-TDE incubation, post-BABB incubation, and post-CUBIC incubation melanoma xenograft samples (data represent mean ± SEM, * *p* < 0.001, one-way ANOVA plus Bonferroni post-test).

The xenograft melanoma samples were cleared using the standard CUBIC protocol (Figure 6), which was optimized to achieve maximum transparency in the least amount of time. As with the human melanoma samples, CUBIC provided significantly better results than the other methods (Figure 7).

BABB and TDE both showed relatively satisfactory results. Still, neither reached the performance of CUBIC. BABB caused a noticeable reduction in size and crumpling of the sample, which, coupled with its incompatibility with certain IHC assays, made it inadequate for fluorescence microscopy. TDE is compatible with IHC, requires shorter preparation times, and is easier to handle, becoming a potentially good alternative to CUBIC when time is a crucial factor.

### 3.3. Hematoxylin & Eosin Staining and TEM Imaging of Cleared Human Melanoma

Previous studies have shown that CUBIC is compatible with H&E staining and that it preserves tissue structure [41]. In this study, we could not perform a quantitative histological comparison between cleared and uncleared tissue due to the limited availability of samples and the fact that the staining rendered the sample unsuitable for posterior optical clearing, therefore impeding direct comparison of the same sample before and after clearing. Therefore, the images were assessed for significant differences in overall tissue structure that could result in skewed or aberrant morphology.

**Figure 8:**
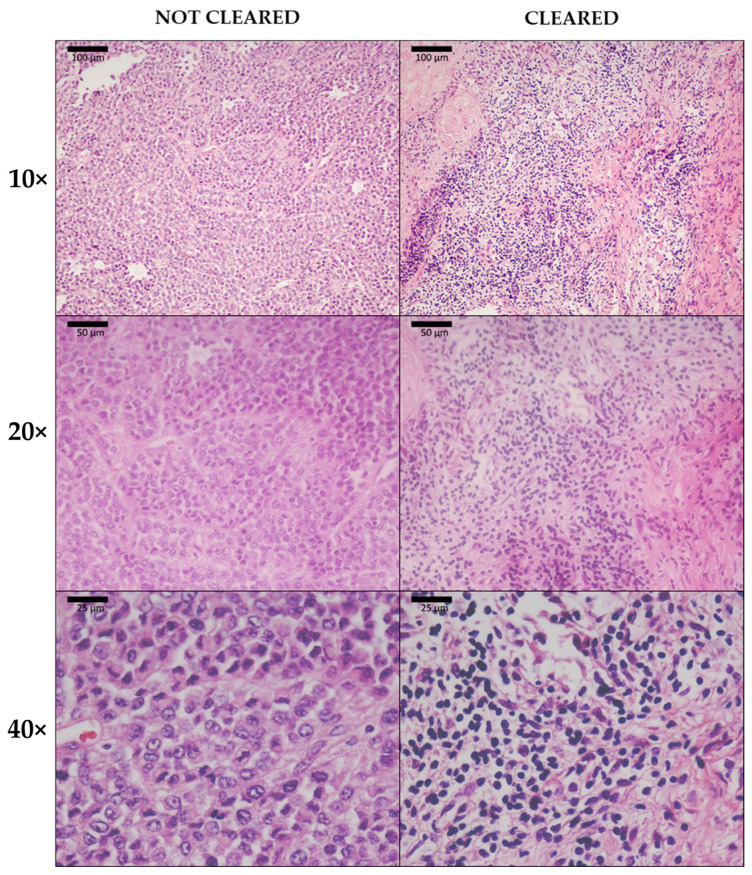
Hematoxylin and eosin (H&E) staining of uncleared tissue (**left**) and CUBIC-cleared tissue (**right**) in 10×, 20× and 40× magnification. The window/level was adjusted uniformly on all images for better visualization.

Upon observation, the structure of the optically cleared tissue itself remained mostly untouched. Some minor differences were present, such as a stronger nuclear tint in cleared samples, which may be caused by an increased cellular permeability to hematoxylin due to CUBIC’s lipid removal. These results confirm CUBIC-cleared tissues as a valuable source of information on tissue structure (Figure 8).

**Figure 9:**
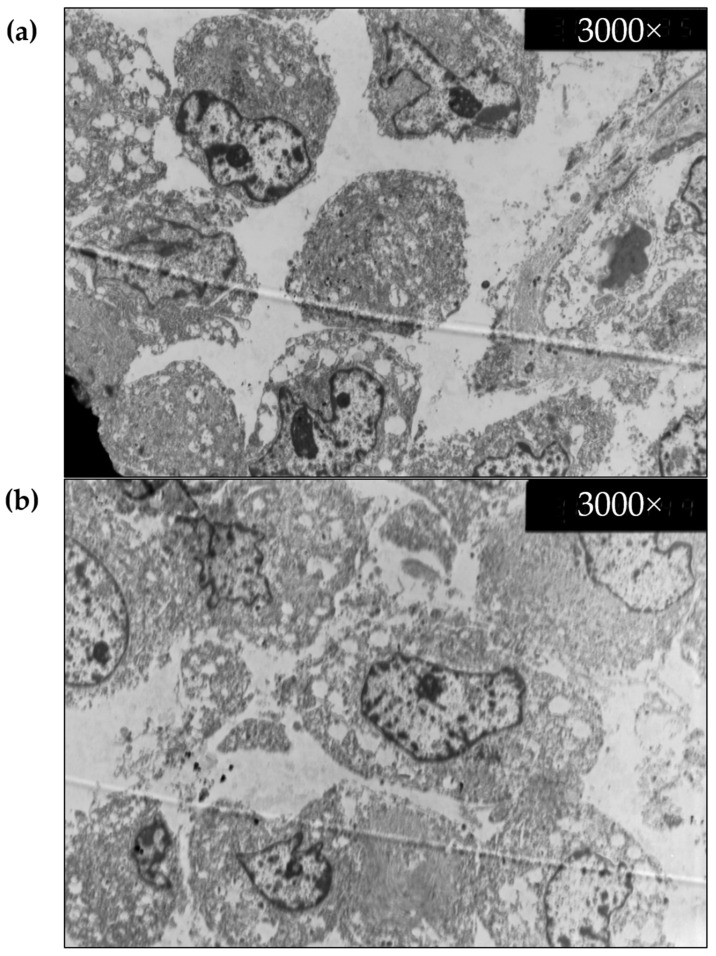
Transmission electron microscopy images of uncleared human melanoma samples, (**a**) uncleared, and (**b**) CUBIC-cleared, 3000× magnification.

TEM microscopy images were successfully acquired on the cleared samples (Figure 9). We noted some differences compared to uncleared samples, such as damage to cellular membranes and an increase in empty vesicles and spaces in the cytoplasm. Whether this was caused by CUBIC’s lipid removal or by other external factors (such as the fact that different regions were taken for each image) could not be assessed with certainty. Nevertheless, it can be said that the overall cellular structure remained similar.

### 3.4. Confocal Cell Population Visualization in Cleared Human Melanoma and Melanoma Xenograft Model

**Figure 10:**
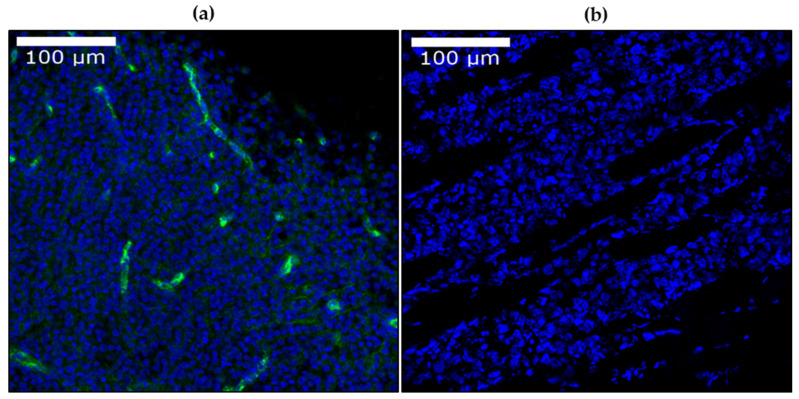
(**a**) Combined results of DAPI (blue) and Factor VIII (green) IHC labelling in CUBIC 1-A cleared human melanoma samples, 20× magnification with confocal microscopy. (**b**) DAPI (blue) IHC labelling in CUBIC cleared melanoma xenograft model, 20× magnification. The window/level was adjusted uniformly on all images for better visualization.

Confocal microscopy images were acquired on the cleared human melanoma samples to assess the compatibility of CUBIC 1-A with IHC. The image content and quality was similar to that of standard confocal images. IHC assays were successful: DAPI labelled the nuclei of all cells, while Factor VIII was observed to be present throughout the length of the blood vessels, displaying their layout and structure (Figure 10a).

Confocal microscopy images were also acquired on the CUBIC-cleared xenograft samples, with the same results. IHC assays were performed to identify cell nuclei with DAPI. These were successful, and images were acquired accordingly (Figure 10b).

### 3.5. SPIM Imaging of Human Melanoma Vasculature and Xenograft Melanoma Model Vasculature

**Figure 11:**
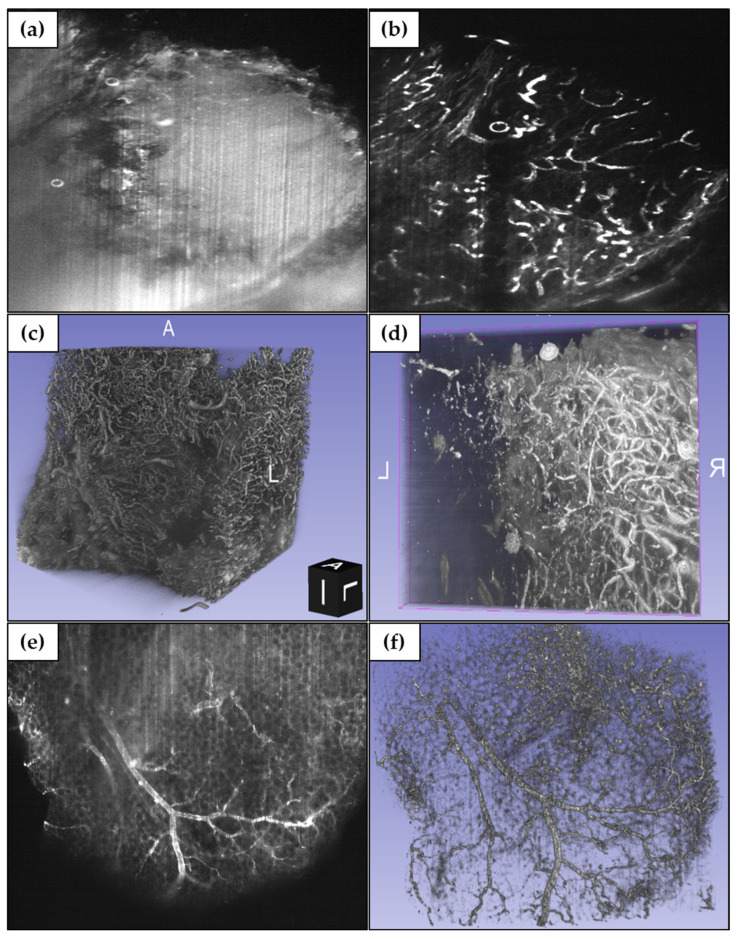
(**a**,**b**) SPIM images of (**a**) DAPI staining and (**b**) Factor VIII staining, 5× magnification. (**c**,**d**) Three-dimensional reconstructions of vasculature structure from acquired SPIM images, (**c**) 5×, and (**d**) 10× magnification. (**e**) SPIM images of cleared xenograft melanoma model, Factor VIII staining, 5× magnification. (**f**) Three-dimensional reconstruction of cleared xenograft melanoma model sample vasculature structure from acquired SPIM images, 5× magnification. The window/level was adjusted uniformly on all images for better visualization.

SPIM images of human melanoma were successfully acquired, with full penetration achieved throughout the whole sample, along with remarkable antibody penetration (Figure 11a,b). No additional differences between superficial areas and deep regions were observed than those expected under similar circumstances. No major illumination artefacts or unevenly lighted areas were found, and the signal depth was only limited by antibody penetration (which was satisfactory, but not absolute, as endogenous labelling would be).

A mild “stripes” artefact (resulting from opaque microparticles) was present in the DAPI channel (405/475 nm); likewise, autofluorescence was observed. Contrarily, the Factor VIII channel (635/670 nm) did not present stripes or autofluorescence, and it showed a better signal to noise ratio (SNR) than DAPI. Thus, long-wavelength dyes can be used for studies that require high-quality 3D images, as opposed to dyes of lower wavelengths.

The high quality of Factor VIII images meant that no further processing was required, outside of minor adjustments for visualization. We successfully processed DAPI images with the use of VSNR and Fiji, and reconstructed the acquired stacks with 3D Slicer. We obtained a detailed model of the vasculature of the sample, which showcased a high vascularization throughout the whole tumor (Figure 11c,d).

Stack size was limited by acquisition time and file size constraints and was kept down to a few millimeters, but continuous stacks spanning the whole sample could be acquired to provide a large-scale atlas of the vasculature of the samples.

By means of a more in-depth analysis, our 3D model can provide information on the distribution of tumoral angiogenesis, and it holds the potential to become relevant in further investigations. It is possible to apply the basics of this project to similar tumoral structures, such as the distribution of the extracellular matrix.

SPIM images of xenograft melanoma model samples were also successfully acquired (Figure 11e), with the same optimal results. Three-dimensional reconstruction of the vasculature was likewise conducted (Figure 11f).

## 4. Conclusions

Here, we establish a novel optimized clearing and labelling protocol for human amelanotic melanoma biopsy samples, and human cell-line derived xenograft melanoma models. The optimized CUBIC protocol can reduce light scattering and increase imaging depth, contrast, and resolution, while remaining compatible with immunohistochemistry assays. This enables its use in combination with 3D imaging techniques for histopathological applications. Using xenograft model samples provides comparable results to those obtained from human clinical samples, while offering a more straightforward acquisition process and a potentially dependable supply.

The possibility of applying this method to other kinds of tumor samples and tissues would have to be examined on a per-tissue basis, as the specific characteristics and molecular compositions will have varying effects on the effectiveness of the CUBIC clearing protocol and the IHC process itself. In the case of more common types of melanoma, the main obstacle to transparency is melanin, which is a light-absorbing pigment. Depigmentation or “bleaching” methods for the histopathological study of melanin-heavy samples do exist, which could pave the way for using these techniques, if no interference or incompatibilities are found between them.

Our findings showcase how the use of optical tissue clearing and advanced 3D imaging techniques can allow for the visualization of vasculature architecture and tumorigenic cell populations in large-scale melanoma samples, potentially providing valuable information on cell dynamics, distribution, and tumor structure that would be absent or significantly altered in traditional 2D H&E pathological studies, or even other 3D imaging techniques with lower depth and/or resolution.

## Figures and Tables

**Table 1:** Reagent composition for CUBIC protocol for human melanoma.

Components	Reagent 1	Reagent 1-A	Reagent 2
N,N,N,N-tetrakis(2-hydroxypropyl)ehylenediamine	20% w/v	5% w/v	-
Distilled Water	40% w/v	75% w/v	15% w/v
Urea	25% w/v	10% w/v	25% w/v
Triton X-100	15% w/v	10% w/v	-
NaCl	-	25 mM	-
Sucrose	-	-	50% w/v
Triethanolamine	-	-	10% w/v

## Data Availability

The data presented in this study are available on request from the corresponding author(s).

## References

[1] Siegel R.L., Miller K.D., Fuchs H.E., Jemal A. (2022). Cancer Statistics, 2022. CA Cancer J. Clin..

[2] World Health Organization (2020). International Agency for Research on Cancer Global Cancer Today.

[3] Almagro J., Messal H.A., Zaw Thin M., van Rheenen J., Behrens A. (2021). Tissue Clearing to Examine Tumour Complexity in Three Dimensions. Nat. Rev. Cancer.

[4] Zhou H., Bacci T., Freund K.B., Wang R.K. (2021). Three-Dimensional Segmentation and Depth-Encoded Visualization of Choroidal Vasculature Using Swept-Source Optical Coherence Tomography. Exp. Biol. Med..

[5] Seo J., Choe M., Kim S.Y. (2016). Clearing and Labeling Techniques for Large-Scale Biological Tissues. Mol. Cells.

[6] Xu J., Ma Y., Yu T., Zhu D. (2019). Quantitative Assessment of Optical Clearing Methods in Various Intact Mouse Organs. J. Biophoton..

[7] Spalteholz W. (1914). Über Das Durchsichtigmachen von Menschlichen und Tierischen Präparaten und Seine Theoretischen Bedingungen, Nebst Anhang: Über Knochenfärbung.

[8] Huisken J., Swoger J., Del Bene F., Wittbrodt J., Stelzer E.H.K. (2004). Optical Sectioning Deep inside Live Embryos by Selective Plane Illumination Microscopy. Science.

[9] Ueda H.R., Ertürk A., Chung K., Gradinaru V., Chédotal A., Tomancak P., Keller P.J. (2020). Tissue Clearing and Its Applications in Neuroscience. Nat. Rev. Neurosci..

[10] Dent J.A., Polson A.G., Klymkowsky M.W. (1989). A Whole-Mount Immunocytochemical Analysis of the Expression of the Intermediate Filament Protein Vimentin in Xenopus. Development.

[11] Dodt H.U., Leischner U., Schierloh A., Jährling N., Mauch C.P., Deininger K., Deussing J.M., Eder M., Zieglgänsberger W., Becker K. (2007). Ultramicroscopy: Three-Dimensional Visualization of Neuronal Networks in the Whole Mouse Brain. Nat. Methods.

[12] Tainaka K., Kubota S.I., Suyama T.Q., Susaki E.A., Perrin D., Ukai-Tadenuma M., Ukai H., Ueda H.R. (2014). Whole-Body Imaging with Single-Cell Resolution by Tissue Decolorization. Cell.

[13] Nehrhoff I., Bocancea D., Vaquero J., Vaquero J.J., Ripoll J., Desco M., Gómez-Gaviro M.V. (2016). 3D Imaging in CUBIC-Cleared Mouse Heart Tissue: Going Deeper. Biomed. Opt. Express.

[14] Nehrhoff I., Ripoll J., Samaniego R., Desco M., Gómez-Gaviro M.V. (2017). Looking inside the Heart: A See-through View of the Vascular Tree. Biomed. Opt. Express.

[15] Gómez-Gaviro M.V., Balaban E., Bocancea D., Lorrio M.T., Pompeiano M., Desco M., Ripoll J., Vaquero J.J. (2017). Optimized CUBIC Protocol for Three-Dimensional Imaging of Chicken Embryos at Single-Cell Resolution. Development.

[16] Shi R., Guo L., Zhang C., Feng W., Li P., Ding Z., Zhu D. (2017). A Useful Way to Develop Effective in Vivo Skin Optical Clearing Agents. J. Biophoton..

[17] Feng W., Shi R., Ma N., Tuchina D.K., Tuchin V.V., Zhu D. (2016). Skin Optical Clearing Potential of Disaccharides. J. Biomed. Opt..

[18] Liopo A., Su R., Tsyboulski D.A., Oraevsky A.A. (2016). Optical Clearing of Skin Enhanced with Hyaluronic Acid for Increased Contrast of Optoacoustic Imaging. J. Biomed. Opt..

[19] Gómez-Gaviro M.V., Sanderson D., Ripoll J., Desco M. (2020). Biomedical Applications of Tissue Clearing and Three-Dimensional Imaging in Health and Disease. iScience.

[20] Sdobnov A.Y., Darvin M.E., Genina E.A., Bashkatov A.N., Lademann J., Tuchin V.V. (2018). Recent Progress in Tissue Optical Clearing for Spectroscopic Application. Spectrochim. Acta—Part A Mol. Biomol. Spectrosc..

[21] Abadie S., Jardet C., Colombelli J., Chaput B., David A., Grolleau J.L., Bedos P., Lobjois V., Descargues P., Rouquette J. (2018). 3D Imaging of Cleared Human Skin Biopsies Using Light-Sheet Microscopy: A New Way to Visualize in-Depth Skin Structure. Ski. Res. Technol..

[22] Sdobnov A., Darvin M.E., Lademann J., Tuchin V. (2017). A Comparative Study of Ex Vivo Skin Optical Clearing Using Two-Photon Microscopy. J. Biophoton..

[23] Idelson C.R., Vogt W.C., King-Casas B., Laconte S.M., Rylander C.G. (2015). Effect of Mechanical Optical Clearing on Near-Infrared Spectroscopy. Lasers Surg. Med..

[24] Levine A., Wang K., Markowitz O. (2017). Optical Coherence Tomography in the Diagnosis of Skin Cancer. Dermatol. Clin..

[25] Ulrich M., Themstrup L., De Carvalho N., Manfredi M., Grana C., Ciardo S., Kästle R., Holmes J., Whitehead R., Jemec G.B.E. (2016). Dynamic Optical Coherence Tomography in Dermatology. Dermatology.

[26] Rajabi-Estarabadi A., Bittar J.M., Zheng C., Nascimento V., Camacho I., Feun L.G., Nasiriavanaki M., Kunz M., Nouri K. (2019). Optical Coherence Tomography Imaging of Melanoma Skin Cancer. Lasers Med. Sci..

[27] Patel J.K., Konda S., Perez O.A., Amini S., Elgart G., Berman B. (2008). Newer Technologies/Techniques and Tools in the Diagnosis of Melanoma. Eur. J. Dermatol..

[28] Pires L., Demidov V., Vitkin I.A., Bagnato V., Kurachi C., Wilson B.C. (2016). Optical Clearing of Melanoma in Vivo: Characterization by Diffuse Reflectance Spectroscopy and Optical Coherence Tomography. J. Biomed. Opt..

[29] Themstrup L., Pellacani G., Welzel J., Holmes J., Jemec G.B.E., Ulrich M. (2017). In Vivo Microvascular Imaging of Cutaneous Actinic Keratosis, Bowen’s Disease and Squamous Cell Carcinoma Using Dynamic Optical Coherence Tomography. J. Eur. Acad. Dermatol. Venereol..

[30] Enfield J., McGrath J., Daly S.M., Leahy M. (2016). Enhanced in Vivo Visualization of the Microcirculation by Topical Application of Fructose Solution Confirmed with Correlation Mapping Optical Coherence Tomography. J. Biomed. Opt..

[31] Feng W. (2018). Visualization of Skin Microvascular Dysfunction of Type 1 Diabetic Mice Using in Vivo Skin Optical Clearing Method. J. Biomed. Opt..

[32] Swetter S.M., Tsao H., Bichakjian C.K., Curiel-Lewandrowski C., Elder D.E., Gershenwald J.E., Guild V., Grant-Kels J.M., Halpern A.C., Johnson T.M. (2019). Guidelines of Care for the Management of Primary Cutaneous Melanoma. J. Am. Acad. Dermatol..

[33] Georges L.M.C., De Wever O., Galván J.A., Dawson H., Lugli A., Demetter P., Zlobec I. (2019). Cell Line Derived Xenograft Mouse Models Are a Suitable in Vivo Model for Studying Tumor Budding in Colorectal Cancer. Front. Med..

[34] Rebecca V.W., Somasundaram R., Herlyn M. (2020). Pre-Clinical Modeling of Cutaneous Melanoma. Nat. Commun..

[35] Samaniego R., Gutierrez-Gonz alez A., Gutierrez-Seijo A., Sanchez-Gregorio S., García-Gimenez J., Mercader E., Marquez-Rodas I., Aviles J.A., Relloso M., Sanchez-Mateos P. (2018). CCL20 Expression by Tumor-Associated Macrophages Predicts Progression of Human Primary Cutaneous Melanoma. Cancer Immunol. Res..

[36] Samaniego R., Estecha A., Relloso M., Longo N., Escat J.L., Longo-Imedio I., Avilés J.A., Del Pozo M.Á., Puig-Kröger A., Sánchez-Mateos P. (2013). Mesenchymal Contribution to Recruitment, Infiltration, and Positioning of Leukocytes in Human Melanoma Tissues. J. Invest. Dermatol..

[37] Ueda H.R., Susaki E.A. (2016). The CUBIC Clearing Protocol with Reagent-1A (for Whole Mouse Brain).

[38] Becker K., Jährling N., Saghafi S., Weiler R., Dodt H.U. (2012). Chemical Clearing and Dehydration of GFP Expressing Mouse Brains. PLoS ONE.

[39] Schindelin J., Arganda-Carreras I., Frise E., Kaynig V., Longair M., Pietzsch T., Preibisch S., Rueden C., Saalfeld S., Schmid B. (2012). Fiji: An Open-Source Platform for Biological-Image Analysis. Nat. Methods.

[40] Fedorov A., Beichel R., Kalpathy-Cramer J., Finet J., Fillion-Robin J.C., Pujol S., Bauer C., Jennings D., Fennessy F., Sonka M. (2012). 3D Slicer as an Image Computing Platform for the Quantitative Imaging Network. Magn. Reson. Imaging.

[41] Matryba P., Sosnowska A., Wolny A., Bozycki L., Greig A., Grzybowski J., Stefaniuk M., Nowis D., Gołąb J. (2020). Systematic Evaluation of Chemically Distinct Tissue Optical Clearing Techniques in Murine Lymph Nodes. J. Immunol..

